# Host-specific co-evolution likely driven by diet in *Buchnera aphidicola*

**DOI:** 10.1186/s12864-024-10045-3

**Published:** 2024-02-08

**Authors:** N. Francois V. Burger, Vittorio F. Nicolis, Anna-Maria Botha

**Affiliations:** Department of Genetics, University of Stellenbosch, Stellenbosch, 7601 South Africa

**Keywords:** Endosymbionts, Host feeding, *Buchnera aphidicola*, *Diuraphis noxia*, Genome structure and gene conservation, Composite strains

## Abstract

**Background:**

Russian wheat aphid (*Diuraphis noxia* Kurd.) is a severe pest to wheat, and even though resistance varieties are available to curb this pest, they are becoming obsolete with the development of new virulent aphid populations. Unlike many other aphids, *D noxia* only harbours a single endosymbiont, *Buchnera aphidicola*. Considering the importance of *Buchnera*, this study aimed to elucidate commonalities and dissimilarities between various hosts, to better understand its distinctiveness within its symbiotic relationship with *D. noxia*. To do so, the genome of the *D. noxia*’s *Buchnera* was assembled and compared to those of other aphid species that feed on diverse host species.

**Results:**

The overall importance of several features such as gene length and percentage GC content was found to be critical for the maintenance of *Buchnera* genes when compared to their closest free-living relative, *Escherichia coli*. *Buchnera* protein coding genes were found to have percentage GC contents that tended towards a mean of ~ 26% which had strong correlation to their identity to their *E. coli* homologs. Several SNPs were identified between different aphid populations and multiple isolates of *Buchnera* were confirmed in single aphids.

**Conclusions:**

Establishing the strong correlation of percentage GC content of protein coding genes and gene identity will allow for identifying which genes will be lost in the continually shrinking *Buchnera* genome. This is also the first report of a parthenogenically reproducing aphid that hosts multiple *Buchnera* strains in a single aphid, raising questions regarding the benefits of maintaining multiple strains. We also found preliminary evidence for post-transcriptional regulation of *Buchnera* genes in the form of polyadenylation.

**Supplementary Information:**

The online version contains supplementary material available at 10.1186/s12864-024-10045-3.

## Background

Phloem feeding aphids occupy a nutritionally constrained niche where plant phloem serves as their sole food source. However, plants vary widely in the constituents and nutritional value of their phloem sap with phloem being rich in carbohydrates and free non-essential amino acids but lacking in complex proteinaceous compounds and free essential amino acids [1]. This imbalance in nutritional proteinaceous compounds and essential amino acids presents challenges to phloem feeding aphids. As growth in hemimetabolous insects, such as aphids, requires intermittent shedding of the exoskeleton when they progress to the next developmental phase, especially chitin production is a continual drain on its internal proteinaceous stocks [2]. The feeding process is also draining as the protective sheath formed from the gelling saliva of aphids is rich in proteins [3]. The protective sheath extends along the aphid’s stylet [4] when the aphid probes different tissue types to assess its nutritional quality and is readily abandoned when feeding sites change to avoid occluded phloem channels [5, 6]. Excreted honeydew has also been shown to contain a diverse set of proteins and free amino acids [7, 8] and is constantly produced to rid the aphid of excess carbohydrates whilst feeding. This expenditure of proteins concurrently happens whilst the parthenogenic aphid rears her developing daughters and granddaughters while also maintaining her own metabolic needs [9]. To supplement this nutritional deficit, all aphids have established symbiotic relationships with either endosymbiotic bacteria or fungi to supplement their nutritional requirements.

*Buchnera aphidicola*, the only member of the *Buchnera* genus, is an endosymbiont found solely in aphid species. *Buchnera* is housed in specialised structures called bacteriocytes [10] where their main function is the production of essential amino acids. Aphids provide *Buchnera* with nutrients in the form of non-essential amino acids which through selective provisioning by the aphid controls the production of essential amino acids [11], and allowing it to adapt to the nutritional contents of its current host. Fulfilling this need becomes even more important as it was found that *Acyrthosiphon pisum* catabolizes and reconstitutes ingested phloem amino acids before shuttling the precursors to *Buchnera* [12].

*Buchnera* is believed to have established its mutualistic relationship with aphids during the late Permian period [13]. Aphids diversified and started specialising to feed on select host plants after the angiosperm radiation in the Cretaceous period [14], after which aphids developed the ability to either feed on a group of plant families (“generalist” or polyphagous) [15] or specialised to feed on a single plant family, such as the Cedar aphid, *Cinara cedri* (“specialist” or monophagous) [16]. Nearly all modern aphids continue this mutualistic relationship with *Buchnera*, with the exceptions of a few aphids of the tribe Cerataphidini (which have formed a mutualistic relationship with a fungus) [17] and the aphid genus Geopemphigus, which have replaced *Buchnera* with a close relative of the Bacteroidetes phylum [18]. The obligatory relationship between aphids and *Buchnera* is evident, as efforts to culture *Buchnera* cells outside of its aphid host has failed [19] and aphids that have been induced to lose their endosymbiotic bacteria (i.e., become aposymbiotic aphids) have low survival and fecundity rates [20–22].

Since the acquisition of the *Buchnera* ancestor (an Enterobacteriaceae member of the Gammaproteobacteria) by aphids roughly 180 million years ago [13, 23], it is believed to have undergone very limited genome rearrangement and lateral gene transfer. However, *Buchnera* has undergone severe genome reduction, mostly through whole gene loss, at an alternating pace in different host species [24]. This is evident by the loss of the *recA* gene in the recombinase pathway [25, 26] responsible for recombination in free living relatives such as *Escherichia coli*. Whole gene loss is proposed to be driven through the continual acquisition of random mutations (Muller’s ratchet) which eventually leads to the loss of gene function and eventual removal after successive cycles of replication [10]. However, the mechanism whereby these genes are removed is unknown but may be due to the increased cost to fitness of either itself or its host [27].

Unlike facultative endosymbionts, where sexual reproduction within aphids is known to be a method of bacterial establishment [28], *Buchnera* transmission is restricted to maternal inheritance. Colonization of *Buchnera* to developing embryos (at the blastula-stage) occurs when proximally located maternal bacteriocytes exocytose *Buchnera* into the hemocoel, which are then endocytosed into the syncytial cytoplasm of the embryo, and packaged into embryonic bacteriocytes [29].

As the functionality of *Buchnera* is of the utmost importance to most aphid species, quantifying the selective adaptation between different *Buchnera* could possibly assist in understanding the continual adaptation of aphids to their hosts. *Diuraphis noxia* (Kurdjumov), or commonly known as Russian Wheat Aphid (RWA), is a cereal grain aphid pest which is known to be invasive and for developing different biotypes — defined here as morphologically similar populations that gained virulence to their hosts leading to breakdown in resistance [30]. The first introduction RWA into South Africa was reported in 1978 and the first resistant cultivar released in 1993 [31]. Shortly following its uptake by wheat farmers, four more virulent RWA biotypes emerged which are defined by their ability to breakdown a differential set of 11 wheat *Dn* (*Diuraphis noxia)* resistance genes [30, 32]. The process of biotypification still remains unclear, but as several of the wheat *Dn* genes have been shown to function through antibiosis (in where the plant becomes less nutritious for the insect host) [33], its interaction with its sole endosymbiont *B. aphidicola* may potentially play a role in overcoming this type of resistance. To better understand the evolution of *Buchnera,* the aim of this study was to first assemble the genome of *Buchnera* of *D. noxia*, followed by the comparison of its genomic composition with that from other members of the Aphidinae. Lastly, to shed light on the role *Buchnera* may play in the acquisition of host characteristics, genomic comparisons between *Buchnera* from the genealogically linked South African biotypes RWA-SA1, RWA-SAM [34], and RWA-SAM2, that express different levels of virulence to their host plant wheat were also conducted.

## Results

### Genome of *Buchnera aphidicola* from *Diuraphis noxia*

**Fig. 1:**
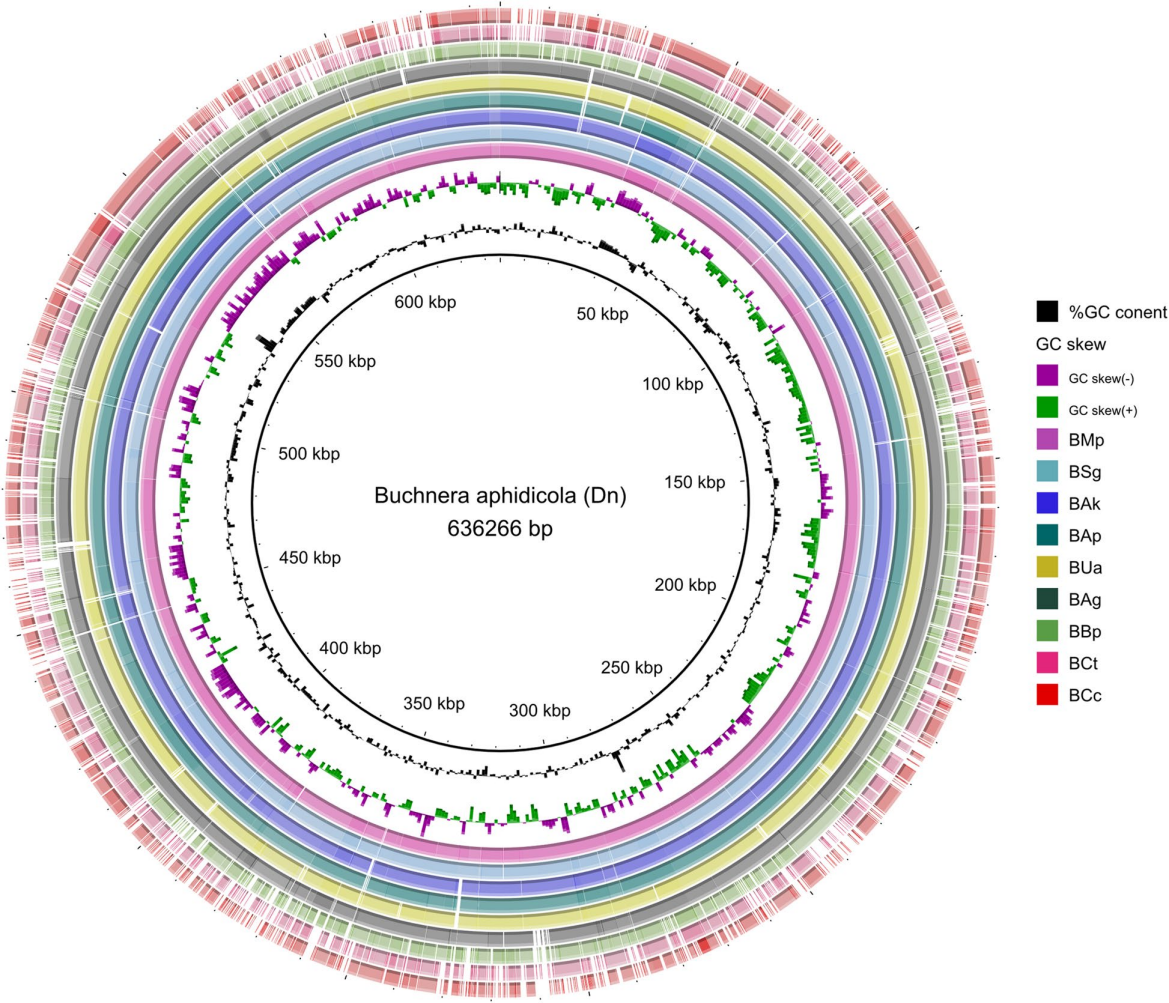
BRIG constructed image showing sequence similarity of *Buchnera aphidicola* from *Diuraphis noxia* (BDn) against that of *Buchnera* from 9 different aphid species

Assembly of *Buchnera aphidicola* from *Diuraphis noxia* (BDn) biotype RWA-SAM produced a genome of 636,266 bp in size, which contains 578 protein coding genes (PCGs), 32 tRNA genes, 3 rRNA genes and 3 other ncRNA genes (total compliment = 616 genes, Table 1, Fig. 1). The average intergenic region between PCGs for BDn is 119.6 bp and average PCG length is 971.6 bp, which is comparable to reported *Buchnera* from various hosts and to that of *E. coli* str. K12. Mapping the entire *D. noxia* Illumina HiSeq NGS dataset to the genome of BDn resulted in respectively 1.35% and 4.18% of the reads for RWA-SAM and RWA-SA1 successfully mapping (Supplementary Table S1).

**Table 1:** Comparison between *Buchnera aphidicola* from 10 aphid species and *Escherichia coli* str. K12

Aphid host	NCBI Accession	Genome size (bp)	Protein coding genes	tRNA genes	rRNA genes	Other RNA genes	Total genes	Avg. protein coding gene length (bp)	Avg. intergenic distance (bp)^a^
*Acyrthosiphon kondoi*	NC_017256	641 794	581	32	3	3	619	972	122.8
*Acyrthosiphon pisum* (Str. 5A)	NC_011833	642 122	579	31	3	3	616	975.1	125.8
*Baizongia pistaciae*	NC_004545	615 980	518	32	3	3	556	969.9	206.7
*Cinara cedri*	NC_008513	416 380	366	31	3	3	403	973	137.2
*Cinara tujafilana*	NC_015662	444 925	393	31	3	3	430	918.7	191.4
*Diuraphis noxia*	NZ_CP013259	636 266	578	32	3	3	616	971.6	119.6
*Myzus persicae* (Str. G002)	NZ_CP002701	643 517	585	32	3	3	623	981.7	109.9
*Schizaphis graminum*	NC_004061	641 454	592	32	3	3	630	962.9	110.7
*Uroleucon ambrosiae*	NC_017259	615 380	546	32	3	3	584	982.2	136.1
*Escherichia coli* str. K-12 substr. MG1655	NC_000913	4 641 652	4 315	86	22	99	4 522	933	132.8

^**a**^ Including distances between protein coding genes and all RNA genes

Comparison of the genic content and genomic features of *Buchnera aphidicola* from 10 different aphid species and its closest free-living relative, *Escherichia coli* str. K12, based on available genome annotation. No pseudogenes were considered in drawing the table

### Genomic synteny and nucleotide identity

**Fig. 2:**
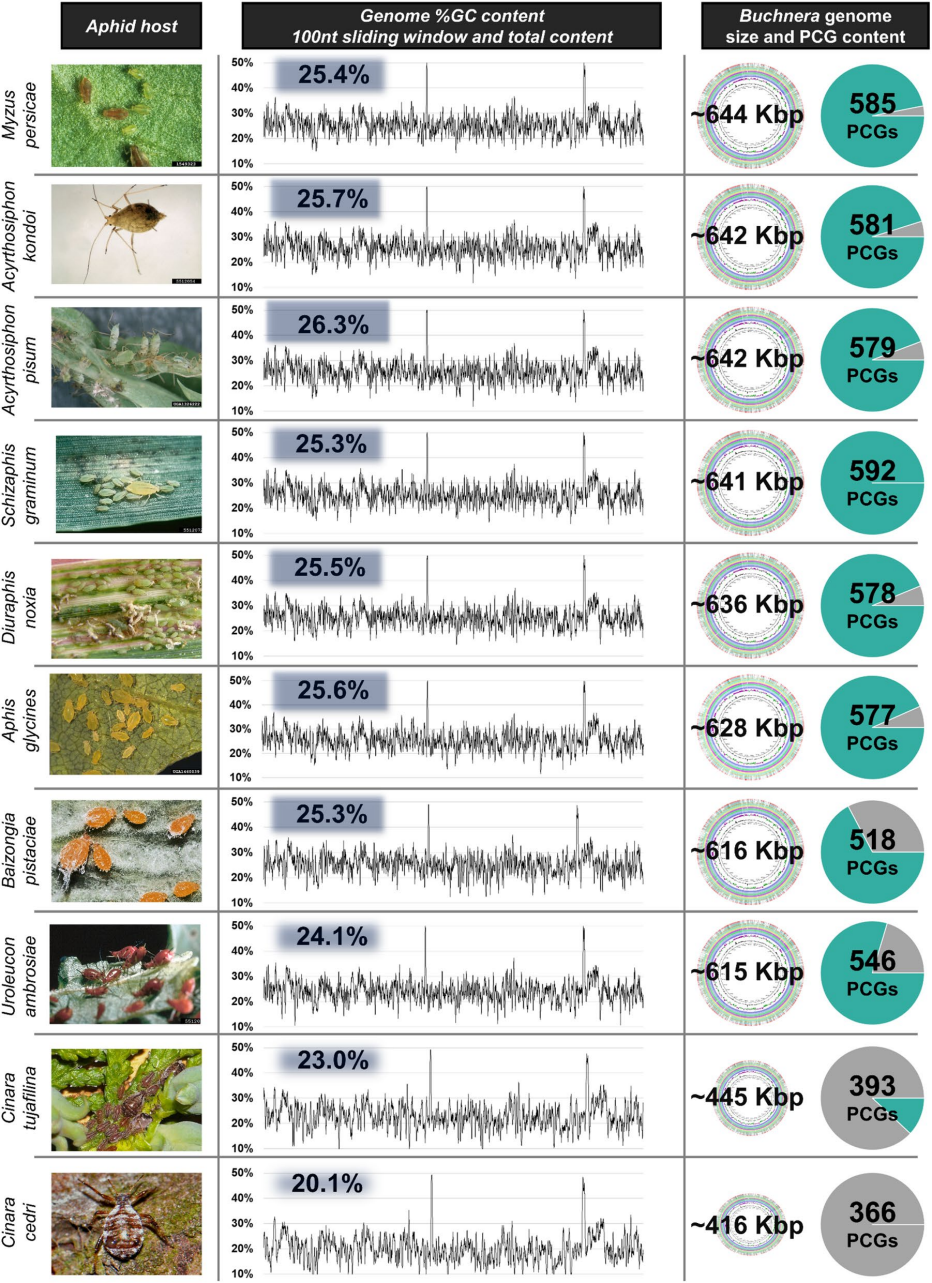
Graphical overview of *Buchnera* host, sliding window genome %GC content, genome total %GC content, genome size and total gene content. Aphid images obtained from: https://www.insectimages.org and https:// influentialpoints.com/

To establish the size and genic content differences between the nine *Buchnera* genomes and BDn, all ten genomes (a total of 5,925,982 bases) were aligned to form a consensus sequence of 715,488 bases. Only 19.2% of bases were similar across all genomes (or 113,779 bases per genome) with an overall average pairwise identity of 59.2%. The pairwise distance matrix obtained from the whole genome alignment (Supplementary Table S2) showed significant variation in nucleotide identities between the genomes that ranged from 84.2% (between BAp and BAk) to 40.8% identity (between BBp and BCc). Overall, the *Buchnera* genomes with nucleotide identities lower than the average were BBp, BCc and BCt. Four of the *Buchnera* genomes in the alignment, originated from shared aphid genera (BAp and BAk from *Acyrthosiphon*; BCc and BCt from *Cinara*) with pairwise nucleotide identities between BAp and BAk equalling 84.2% and that of BCc and BCt equalling only 57.3%. To assess if genomic synteny extends to %GC content, a sliding window assessment of %GC content was performed for all ten *Buchnera* genomes. Overall, the similarity in %GC content across the genomes was found to be similar (Fig. 2) although gene content and genome sizes differed greatly (Table 1).

To assess genomic synteny within the ten *Buchnera* genomes, an array representing the collective genic content was constructed (Additional File 1), as well as a Mauve alignment over the full lengths of the ten *Buchnera* genomes and *E. coli* str, K12 (Supplementary Figure S1). From the Mauve alignment it is clear that no large scale syntenic blocks have rearranged, as has been previously reported [24]. The established gene array consisted of 697 rows, of which 659 rows contained PCGs, 32 rows tRNAs, three rows rRNA genes, two rows ncRNAs and one row with a tmRNA gene. Of the 659 PCG rows, 556 rows were successfully assigned identity towards *E. coli* str. K12, while *E. coli* identities were also assigned to a row if at least one PCG within that row contained a reciprocal match to *E. coli*. This resulted in 90 additional PCG rows obtaining an identity from *E. coli* str. K12, from which two gave no reciprocal matches due to gene duplications in the *Buchnera* genomes (*grpE* and *trpD*), 34 rows due to genes that have been split (25 unique genes split multiple times, Supplementary Table S3), and 64 possibly due to severe deterioration. When compacting the duplicated rows in the array, there are 659 genes (621 PCGs, 32 tRNA, two ncRNA, one tmRNA, and three rRNA genes) in the pan genome of the most recent common ancestor (MRCA) of the *Buchnera* genomes (Table 1), while shared genes amount to 364 (328 PCGs, three rRNA, two ncRNA, one tmRNA and 30 tRNA genes). From the gene array, it is evident that gene synteny was maintained between the different *B. aphidicola* genomes as previously reported [24], with the only deviations being changes in gene direction (one hypothetical gene at position 43 and *pyrF* at position 283 in the gene array in BBp; tRNA-Ser at position 354 in BCc and BCt) and one inversion (ygfZ-prfB-lysS-lysA-lgt-thyA) in BBp at positions 467 to 473. Comparing similarities between the *Buchnera*s’ and *E. coli* str. K12’s genomes it appears that large sections maintained relative gene order and direction, with only 292 *Buchnera* PCGs in the opposite direction of those in *E. coli*. Reconstructing syntenic blocks shared between *E. coli* and the *Buchnera* gene array revealed that the pan genome of *Buchnera* can likely be assembled from 185 *E. coli* syntenic blocks (Additional File 1), with the smallest syntenic blocks containing only 1 gene (61 occurrences), and the largest syntenic block spanning 41 genes (position 529 to 569) and 28 ribosomal genes.

**Fig. 3:**
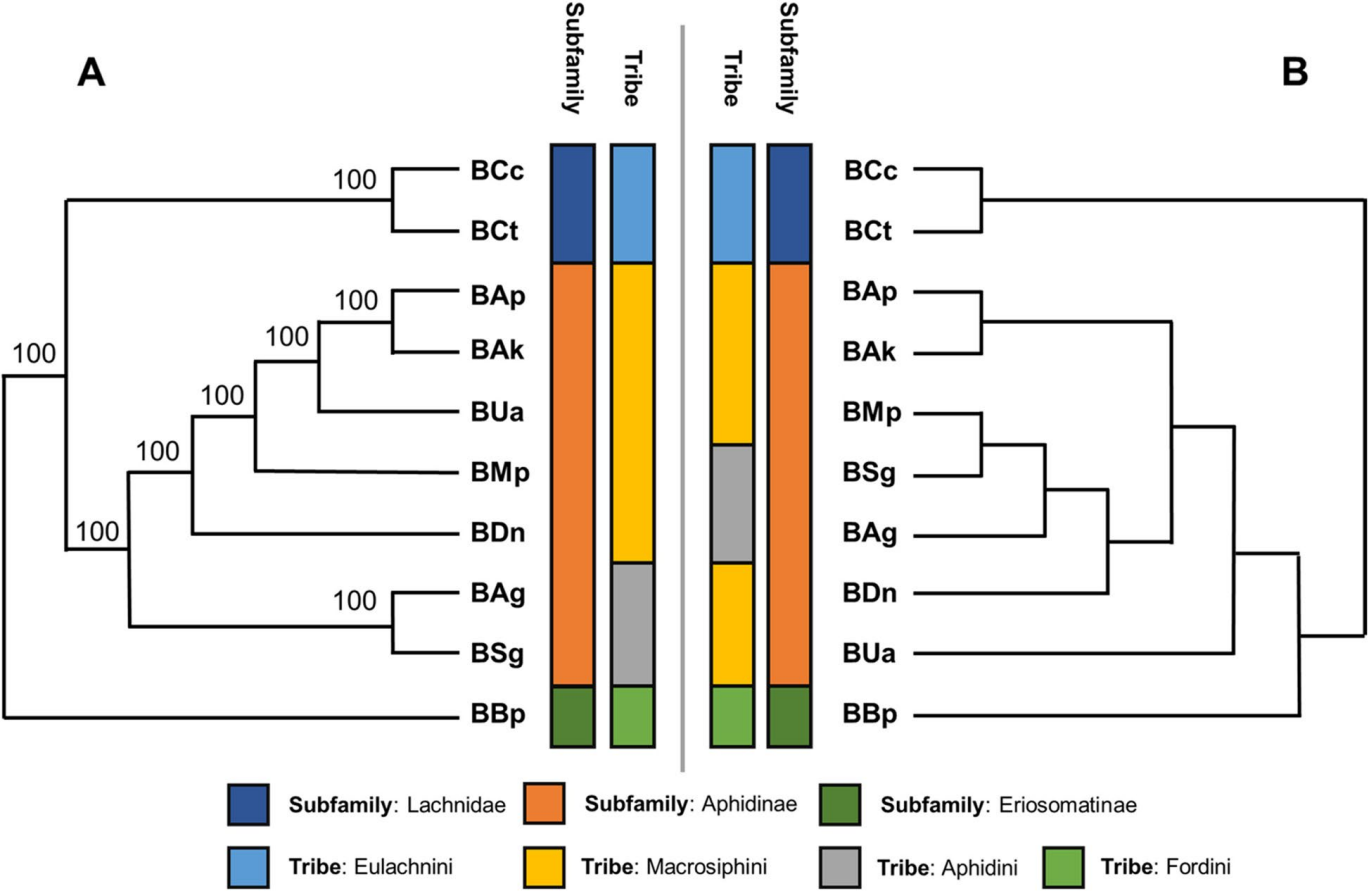
(**A**) PAUP maximum parsimony tree with transformed branches and numbers indicating bootstrap replicate consensus compared to a (**B**) Cluster 3.0 produced dendrogram illustrating shared gene content between the 10 *Buchnera* genomes considered during this study

The obtained gene array was also converted into a binary matrix and analysed to identify shared patterns of gene content between the *Buchnera* genomes. The resulting clustered dendrogram had two main clusters with one containing all members of the subfamily Aphidinae (BAg, BAp, BAk, BDn, BMp, BSg and BUa) and Eriosomatinae (Fordini)—(BBp), and the other members of the family Lachnidae (BCc and BCt). In the Aphidinae subfamily cluster, BUa (Macrosiphini tribe) and BBp (Fordini tribe) were outliers from the inner clusters; where BDn (Macrosiphini tribe) grouped with BAg (Aphidini tribe), BMp (Macrosiphini tribe) and BSg (Aphidini tribe), while BAp and BAk clustered together. Comparing the obtained gene cluster dendrogram with a PAUP generated phylogenetic tree of whole genome alignments (Fig. 3), the similarity in grouping between the trees are noticeable.

### Genic %GC content and homologue protein identity

**Fig. 4:**
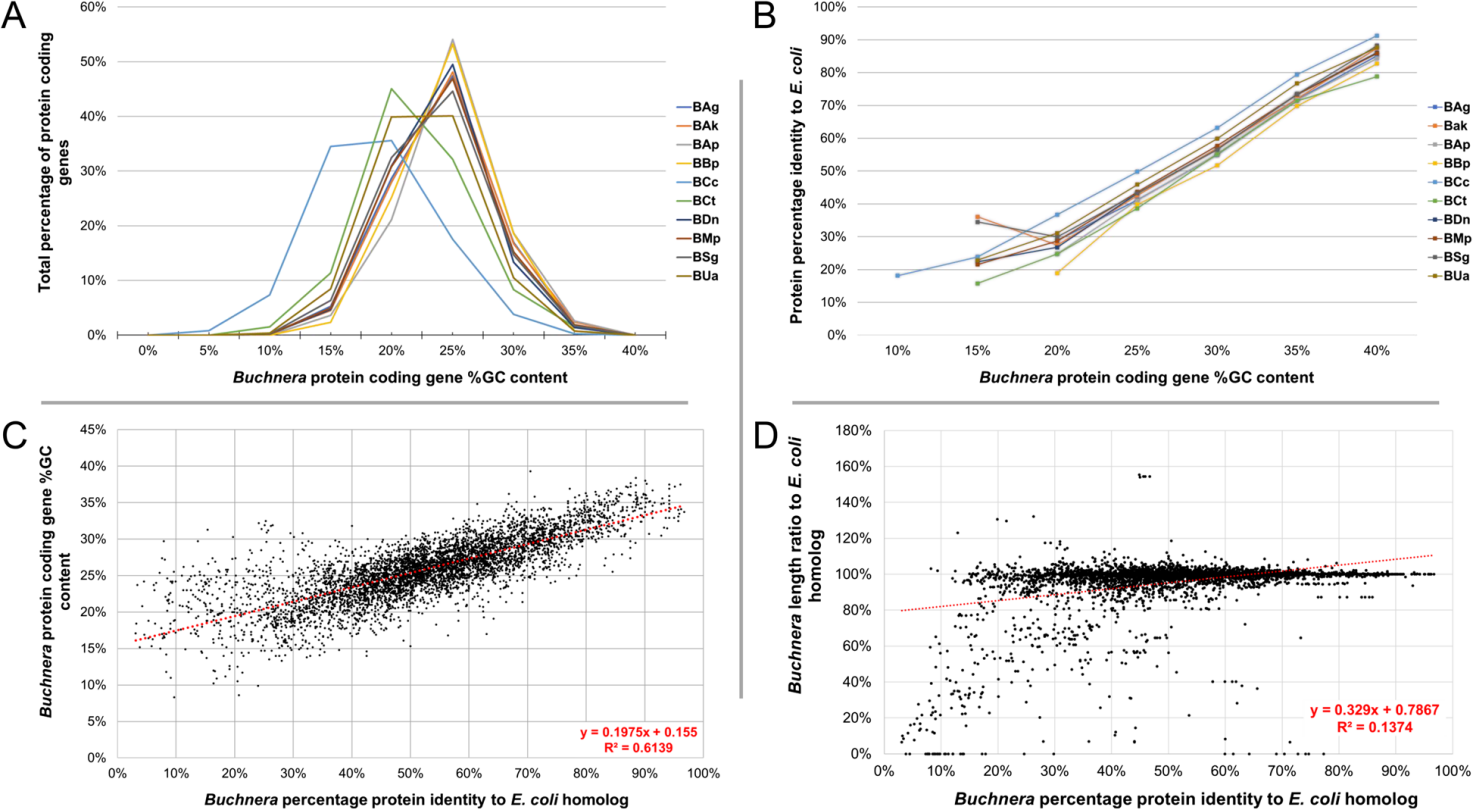
A combined image of (**A**) a line graph illustrating the relative percentage of *Buchnera* genes plotted over their %GC content; **B** A line graph illustrating the averaged PCG %GC content of individual *Buchnera* genomes and their averaged protein identity to *E. coli*; **C** A scatter plot of the %GC content of all 5,508 *Buchnera* PCGs plotted against their percentage protein identity when aligned to their *E. coli* homolog; and (**D**) a scatter plot of all 5,508 *Buchnera* PCG’s relative percentage length when compared to their *E. coli* homolog

For the ten *Buchnera* considered in this study, their PCG %GC content ranged from 39.3% to 8.3% (Fig. 4A, Additional File 1) with a distinct mean around ~ 26%. The only *Buchnera* skewed %GC contents being those of BUa, BCc and BCt (mean %GC content) and tended to the lower ranges. Of these three, PCGs from BCc had the lowest overall %GC content (%GC = 21.22% ± 4.76% SD). The average gene lengths in the genomes were similar (mostly < 5% length variation) (Table 1) with an overall genic length conservation of 95.93% (± 15.85% SD) when compared to the *E. coli* homologs, however, the %GC content conservation corresponded to only 48.90% (± 9.52% SD) (Additional File 1).

On average, *Buchnera* genes shared a percentage protein identity of 52.66% (± 16.6% SD) with their *E. coli* homologs (Additional File S1). Plotting the averaged percentage protein identity of all *Buchnera* PCGs to their *E. coli* homologs against the *Buchnera* PCG %GC content (Fig. 4B) revealed a linear correlation. Individual *Buchnera* PCGs %GC content was also plotted against their individual percentage protein identity of their respective *E. coli* homologs (Fig. 4C) to produce a scatter plot, which also portrayed a linear relationship. Gene length conservation between *Buchnera* PCGs and their respective *E. coli* homolog plotted over PCG percentage protein identity (Fig. 4D) however, did not show such a linear correlation, with 28 genes found to be 10% larger than their *E. coli* homolog.

To determine the contribution of length conservation and %GC PCG content as an indicator for shared protein sequence similarity with *E. coli*, a multiple linear regression model was applied (Additional File 2) to show that both PCG gene length and %GC content significantly contributes towards the PCGs protein identity, with %GC content being the most influential metric (standardized coefficient = 0.784). From the equation of the multiple linear regression model, an increase in 1% of PCG %GC content increases protein identity to *E. coli* by ~ 3.12%.

To establish which genes have maintained their conservation, the scatter plot representing *Buchnera* PCGs %GC content plotted over *E. coli* identity was divided into four quadrants (Q1 – Q4) intersecting both the mean *Buchnera* PCG %GC content (~ 25.86%) and percentage protein identity to *E. coli* (~ 52.66%) of the combined data set (Supplementary Figure S2). To ensure that the mean %GC content of *Buchnera* PCGs were reflective of other *Buchnera* strains that did not form part of this study, the %GC of all annotated *Buchnera* PCGs available on the NCBI (date accessed: 2023/08/01) were also plotted (Supplementary Figure S3) and determined to be 25.69%.

**Fig. 5:**
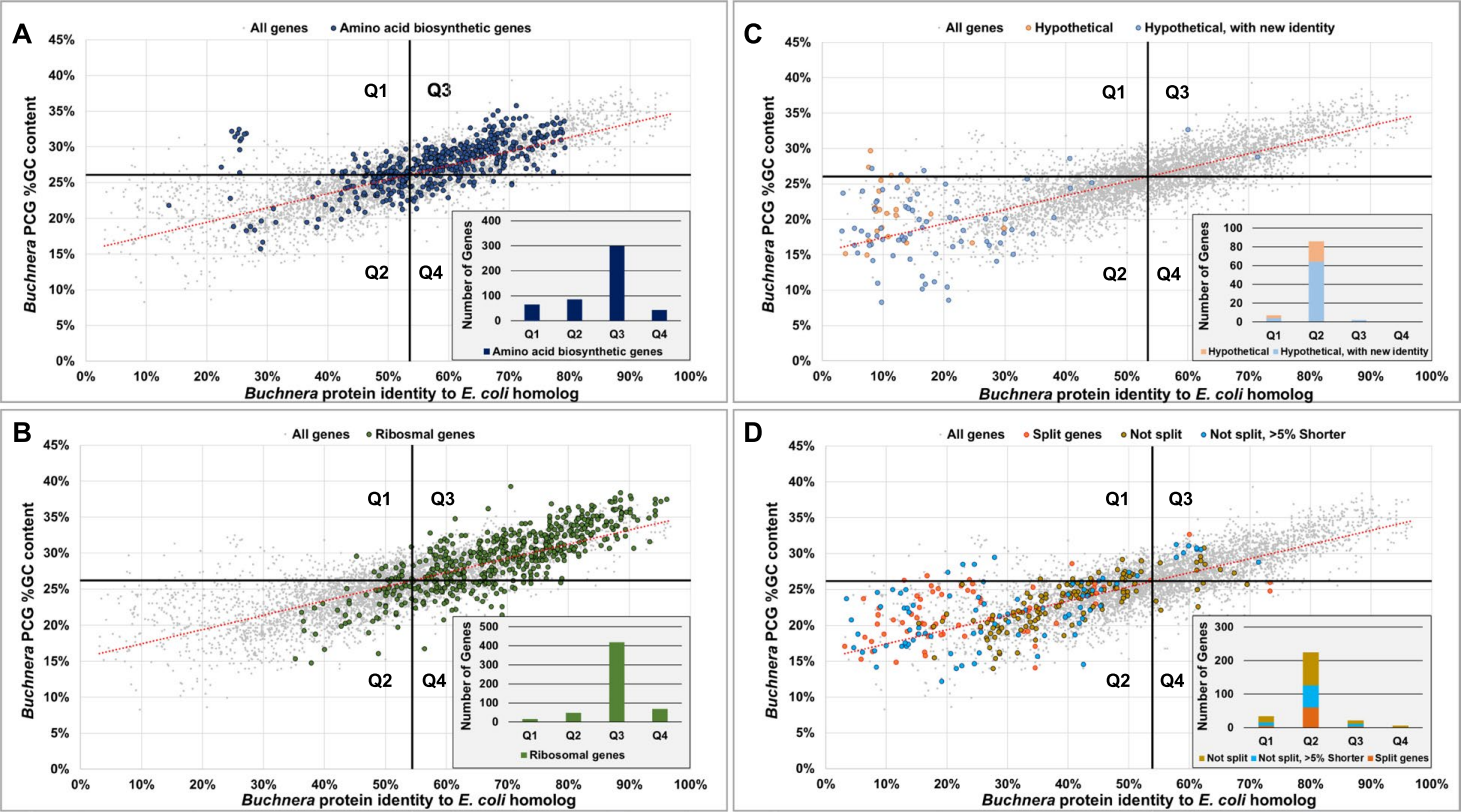
Scatter plots of *Buchnera* PCG %GC contents plotted over their percentage identity to their respective *E. coli *homologs. Overlayed on these scatterplots are (**A**) genes involved in amino acid biosynthesis, (**B**) genes labelled as hypothetical in the various genomes, (**C**) genes encoding for ribosomal proteins, and (**D**) genes that have been identified as split in the current study, or that shares the same row in the constructed gene array. The four quadrants represent PCGs with (Q1) values below the mean percentage protein identity and above the mean %GC content; (Q2) PCGs with values below both the mean %GC and percentage protein identity; (Q3) PCGs with values above both the mean %GC and percentage protein identity; and (Q4) PCGs with values below the mean %GC and values above the mean percentage protein identity

To assess whether the level of conservation could be extrapolated from plotting PCG %GC content over *E. coli* identity, genes that would be expected to be well maintained (those involved in amino acid biosynthesis and all ribosomal genes) and those that are suspected of being lost (genes labelled as hypothetical and all genes that were in the same row in the gene array as those identified as having a split annotation) (Supplementary Table S3), were superimposed over Fig. 4C (Fig. 5). Of the total 491 genes involved in amino acid biosynthesis, the majority plotted in Q3 (299 genes), followed by Q2 (86 genes), then Q1 (64 genes), and the least in Q4 (42 genes) (Fig. 5A). Superimposing the 550 *Buchnera* genes coding for ribosomal subunits followed a similar trend where most fell in Q3 (419), followed by Q4 (69), then Q2 (48), and the least in Q1 (14) (Fig. 5B). Of the 95 genes labelled as hypothetical in the 10 *Buchnera* genomes considered in this study, the majority fell into Q2 (86), followed by Q1 (7), and the least in Q3 (2) with none present in Q4 (Fig. 5C). The 69 *Buchnera* genes with split annotations (and members sharing genes in that row), grouped mostly in Q2 (225), followed by Q1 (34), then Q3 (21), and the least in Q4 (6) (Fig. 5D). To further assess the correlation of GC content to *Buchnera* gene conservancy, 29 genes previously shown to be under positive selection [24] were superimposed over Fig. 4C (Supplementary Figure S4; Additional File 3). Of the total 290 genes (29 from the ten *Buchnera* genomes) present in this study, 140 plotted in Q1, 76 in Q3, 32 in Q2 and 22 in Q4.

### Nucleotide variation within *Buchnera aphidicola* from *Diuraphis noxia* (BDn)

**Fig. 6:**
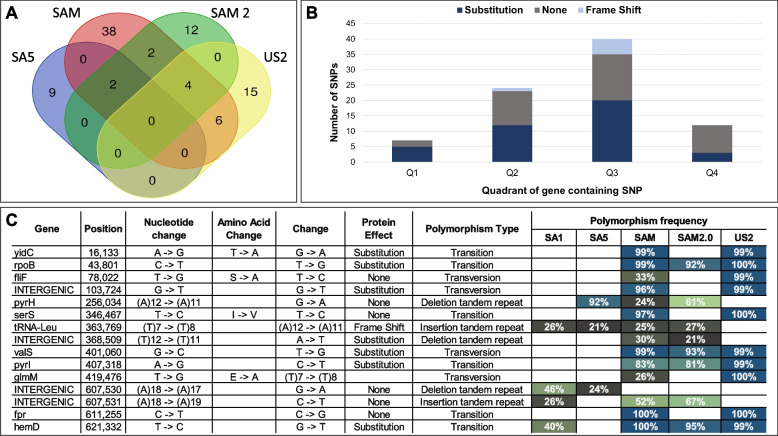
Single nucleotide polymorphisms identified in BDn from NGS sequences of RWA-SA1, RWA-SA5, RWA-SAM, RWA-SAM2 and RWA-US2. **A** A Venn diagram illustrating the number of SNPs shared between RWA-SA5, RWA-SAM, RWA-SAM2 and RWA-US2 when mapped against the BDn genome of RWA-SA1. **B** A bar graph indicating the scatterplot quadrant of SNP containing genes, and (**C**) all BDn SNPs shared between at least two *D. noxia* biotypes tabulated with their observed frequency

To establish the nucleotide variance within BDn, NGS reads from South African *D. noxia* biotypes (RWA-SA1, RWA-SA5, RWA-SAM, and RWA-SAM2) [35] and biotype US2 [36] were mapped to the BDn genome of RWA-SA1 (SUB13877298). A total of 121 nucleotide polymorphisms were identified, of which 39 were shared between at least two biotypes. The *D.* *noxia *biotype with the most identified SNPs was RWA-SAM (with 52 SNPs), followed by RWA-US2 (with 25 SNPs), RWA-SAM2 (with 20 SNPs), RWA-SA1 (with 13 SNPs) and lastly RWA-SA5 (with 11 SNPs) (Fig. 6A; Additional File 4). Of the 121 polymorphisms, 83 were identified in 61 different PCGs and either resulted in an amino acid substitution (40 SNPs), had no protein effect (40 SNPs), or induced a frame shift (6 SNPs). Most genic SNPs were localized in genes that plotted in Q3 (40 SNPs), followed by Q2 (24 SNPs), then Q4 (12 SNPs), and lastly in Q1 (7 SNPs) (Fig. 6B; Additional File 4).

**Fig. 7:**
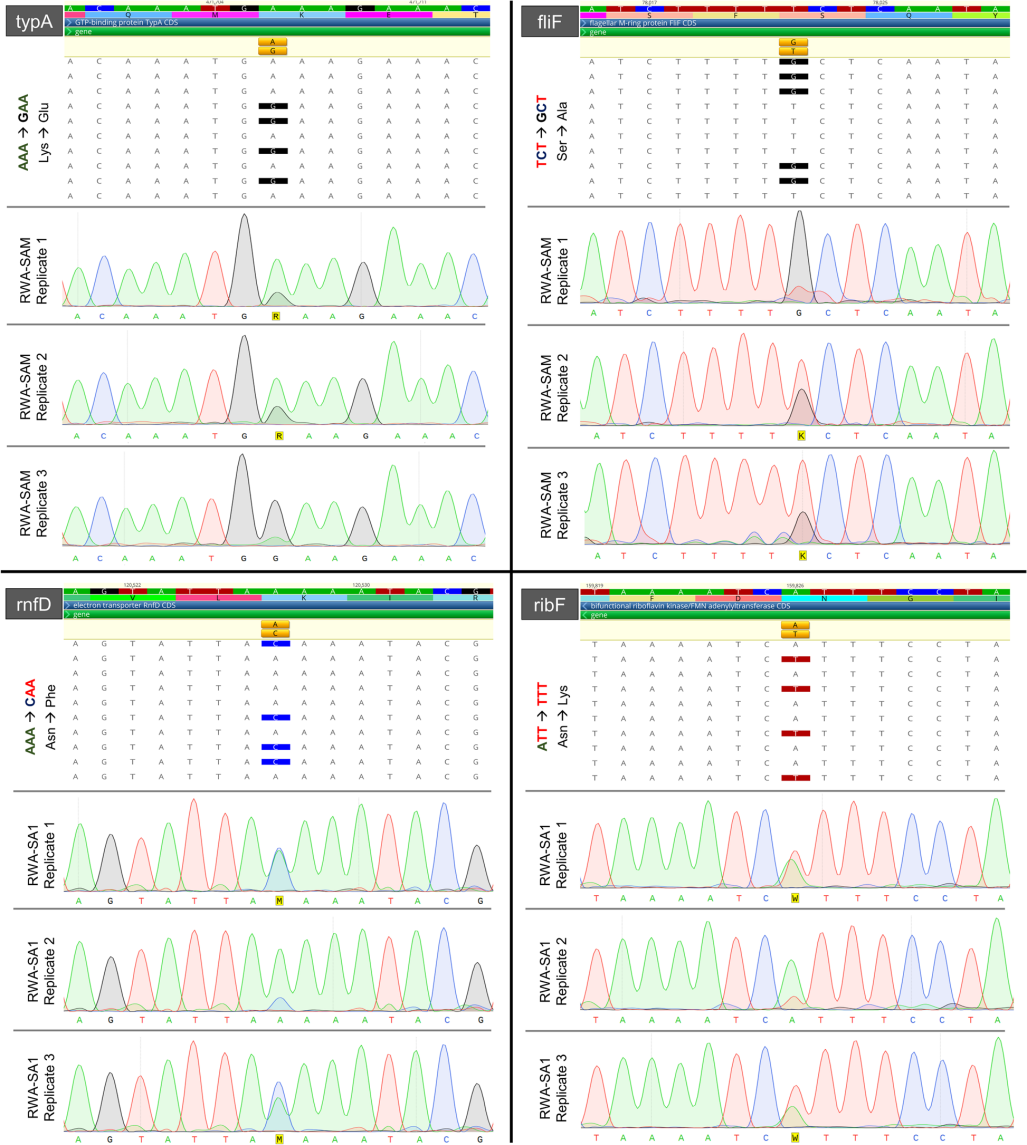
Single nucleotide polymorphisms detected through Sanger sequencing of genomic DNA extractions from single aphids of BDn from various *D. noxia* biotypes. Names and genomic locations of genes sequenced appear above the alignments

When investigating the SNP frequencies versus that of the expected reference bases, several of the polymorphic positions present in the various BDn genomes occurred at frequencies other than 100% (Fig. 6C and Additional File 4). After mapping the sequencing reads back to their individual reference genomes, it was apparent that *Buchnera* from RWA-SAM presented with the most variable polymorphic sites (where SNP frequencies were between 20–80%) with 44 sites, followed by RWA-SA1 (13 variable sites), RWA-SA5 (10 variable sites), RWA-SAM2 (8 variable sites) and lastly RWA-US2 (2 variable sites). To validate the observed variation in SNP frequency, four genic areas showing variable SNP frequency profiles between RWA-SA1, RWA-SAM (Supplementary Table S4) were selected for Sanger sequencing using DNA from single aphid extractions. All variable sites sequenced displayed double chromatogram peaks at the predicted variant positions (Fig. 7). The Gene Ontology profiles of genes that contained SNPs were also compared to ascertain the functions of the SNP containing genes (Supplementary Figure S5), with most GO terms were related to metabolism and cell cycle regulation, and a few involved with response to stress and response to chemicals.

### Investigating expression of BDn transcripts between *D. noxia* biotypes

Previously, de novo assembly of NGS RNA-seq reads from RWA-SA1 and RWA-SAM identified transcripts of *Buchnera* origin [37]. To assess if these transcripts originated from *Buchnera*, instead of an aphid horizontal gene transfer event, RNA-seq reads were mapped to the BDn genome. Mapping individual RNA-seq samples of RWA-SA1 and RWA-SAM onto the BDn genomes resulted in respectively ~ 2.44% and ~ 3.44% of the total RNA-seq reads successfully mapping (Additional File 5). To assess if this mapping ratio was similar to whole aphid RNA-seq which used total RNA as input for library preparation (instead of an mRNA library preparation kit as used in Nicolis et al. [37]), RNA-seq reads from *D. noxia* obtained from the NCBI’s SRA database were mapped to BDn (Additional File 5). Mapping RNA-seq reads originating from RWA-US1 (nine individual single-end read sets from Rojas et al. [33]) identified ~ 1.58% successfully mapped reads, while RNA-seq reads originating from RWA-US2 (two individual paired-end read sets from Nicholson et al. [36]) identified 5.49% and 2.9% reads successfully mapping. To ascertain if the presence of polyadenylated BDn transcripts identified during the in silico analysis were present, and not due to a library construction anomaly, cDNA synthesis was performed using RNA obtained from *D. noxia* SA1 making use of oligo dT_(18)_ primers. Following cDNA synthesis, genes of interest (Supplementary Table S4) were PCR amplified from both the oligo dT_(18)_ and a no-reverse transcriptase (no-RT) control cDNA library and visualised through agarose gel electrophoresis. To ensure that no internal stretches of adenosines were responsible for cDNA amplification, primers were designed on the 3’ ends of the genes of interest. All genes were successfully amplified whilst the no-RT controls only contained primer shadows (Supplementary Figure S6).

To assess if PCG %GC content was a determinant for gene expression (as was previously postulated in Schaber et al. and the citations within [38]), gene expression was categorized as either being low (logCPM < 9), medium (logCPM between 9–11), or highly expressed (logCPM > 11) (Additional File 5). These genes were then superimposed over the scatterplot produced from the %GC content of *Buchnera* PCGs over their protein identity of their *E. coli* homologues (Supplementary Figure S7).

## Discussion

### GC content is conserved on a whole genome and individual gene level between *Buchnera* from distantly related aphids

The close and unique association that *Buchnera* shares with its various hosts has led to an extraordinary 36% variation in its genome size (considering the genomes investigated in this study; ~ 654 Kb, *Myzus persicae* to ~ 422 Kb, *Cinara cedri*). As has been previously established, this variance originates from direct loss of functional genes that remarkably maintains overall gene synteny [24]. Interestingly, there also appears to be an overall conservation of GC content across all the studied *Buchnera* genomes, albeit their varied genome sizes and genic content (Fig. 2). The overall gene structure in *Buchnera* remained very similar across the studied *Buchnera* genomes, and that of their closest free-living relative *Escherichia coli,* with a difference of ~ 10% between the average intergenic distance of *E. coli* genes and the genes of BAk, BAp, BCc, BDn and BUa (Table 1). This was not the case for the intergenic distance for genes of Bag (-15%), BBp (+ 56%), BCt (+ 44%), BMp (-17%), and BSg (-17%). Intergenic distance within *Buchnera* (and Prokaryotes) are important for maintaining gene function as they contain the regulatory elements required for regulation of gene expression, such as Shine-Dalgarno sequences, transcriptional terminators, Sigma-32 binding sites and small RNAs [39–41]. Both BCc and BCt are believed to have recently undergone severe gene loss [42], which may indicate that the increased intergenic spaces observed in their genomes are remnants of these losses. If so, then the increase in intergenic space distance observed in BBp and BCt may be remnants of recently experienced multiple gene losses where they have not yet lost the flanking intergenic DNA.

Another noteworthy finding was the strong linear relationship between the %GC content of *Buchnera* PCGs and their percentage protein identity towards their *E. coli* homologs (Fig. 4C). Plotting *Buchnera* genes that would reasonably be expected to remain conserved (those partaking in the synthesis of amino acids and ribosomal genes) plotted mostly in quadrant 3 (high percentage identity to their *E. coli* homologues and a relatively high %GC content), whilst genes that can reasonably expected to be undergoing purifying selection (genes annotated as hypothetical or that have a split annotation due to internal stop codons) plotted mostly in quadrant 1 (low percentage identity to their *E. coli* homologues and a relatively low %GC content; Figs. 5 A-D). Protein coding genes that were split across two separate annotations in some of the genomes considered, and their non-split syntenic counterparts from other *Buchnera* (as determined by the produced gene array; Additional File 1), were also found to be smaller than their *E. coli* homologues. The strong correlation of PCG %GC content and *E. coli* homologue identity established in this study may explain why the majority of the *Buchnera* had a mean %GC content of ~ 26% (Additional File 1). As it appears that protein coding genes with relatively low %GC contents are being lost from the genomes, the prominent ~ 26% mean %GC content may indicate a threshold line (tipping point) whereby genes that maintain %GC contents at this point or higher are secured for future transmission. The only *Buchnera*s considered in this study that did not follow this %GC conservancy were BCc, BCt and BUa, whose means skewed towards the lower ranges, indicating that they may actively be undergoing a purging of genes. The correlation of high %GC content and gene conservation is supported by findings made in other aphids where it was found that aphid endosymbionts (in both *Buchnera* and other facultative endosymbionts) with larger genomic %GC contents housed more genes [43]. As selection pressure to maintain these genes disappear, loss of evolutionary constraint would eventually lead to the proliferation of AT bases [44]. Interestingly, a recent study [45] found that intrachromosomal elements (such as plasmids) with high AT contents are favoured by their hosts (and so likely endosymbionts as well), most likely due to a lowered metabolic cost for their maintenance. This would help explain the presence of large %GC rich genic areas surrounded by mostly AT rich DNA.

### Different *Buchnera* genotypes are harboured within single aphids

In this study, it was found that *Diuraphis noxia* biotypes, despite reproducing mostly through parthenogenesis, contain more than one *Buchnera* strain (genotype) (Figs. 6 and 7). Since *Buchnera aphidicola* is the sole endosymbiont of *D. noxia*, it was assumed that *Buchnera* would be present as a single genotype, as maternal transfer of *Buchnera* (through exocytosis out of maternal bacteriocytes) to embryos (through endocytosis) is believed to be the only method that *Buchnera* can spread [29]. The earliest estimate of biotypification of *Diuraphis noxia* likely started more than 50 years ago with the radiation of the species from Europe and the fertile Crescent to wheat and barley producing areas on other continents. The best documented settlement of this invasive pest elsewhere was in South Africa in 1978, and in the USA in the early 1980s. Over a period of roughly 30 years, this pest was controlled by pest management strategies such as planting resistant varieties and applying insecticide [30], but several new biotypes were reported from 2006 onwards. It was interesting to note that several of the variants were shared between US and South African aphid populations (15 variant sites; Additional File 4), although they have not been in contact with one another for at least 40 years. As the inheritance of *Buchnera* appears to be restricted to an almost random endocytosis of *Buchnera* cells contained in the extracellular space [29], there may be a fitness advantage to maintaining these alternate strains. A previous study demonstrated a similar ability when a non-native *Buchnera* genotype was successfully transplanted to an aphid, thereby artificially creating an aphid with multiple endosymbiont genotypes [46]. The non-native *Buchnera* genotype contained the *ibpA* allele that confers heat tolerance to the aphid, whilst the native *Buchnera* did not. After successive exposures to heat the non-native *Buchnera* genotype outcompeted the original *Buchnera* after a few generations of its host. Hence, not only it is possible for an aphid host to maintain more than one *Buchnera* genotype, but the beneficial *Buchnera* can prevail/outcompete the less fit *Buchnera*, assisting host adaptation to different environmental cues [46]. Whether though multiple *Buchnera* genotypes facilitate biotype development in *D. noxia* is still unclear and further research is required.

### Expression of *Buchnera *transcripts between *D. noxia* biotypes are relatively stable

When viewing the total expression of BDn genes in RWA-SA1 and RWA-SAM, it appears that more BDn genes are expressed in RWA-SA1 at slightly lower levels than in RWA-SAM. The total number of transcripts required to reach 90% of total expression (Ex90) is three more in BDn of RWA-SA1 than in BDn RWA-SAM (Additional File 5) which is opposite to what was found for the expression of aphid transcripts in RWA-SA1 and RWA-SAM (Supplementary Figure S8) [37] where RWA-SAM had ~ 1,600 more transcripts than RWA-SA1 accounting for 90% of its total expression. Unfortunately, data on *Buchnera* gene expression is conflicting. Gene expression was reported not to differ greatly under extreme environmental conditions [47] or when aphid feeds on diets differing in nutritional content [48], but reported to differ when the host fed on a Leucine depleted diet [49], and during development (adult vs developing embryos [50]. This later was however disputed, as it is believed that *Buchnera* has developed a diminished capacity to regulate its transcription through loss of regulatory elements [40] and canonical regulatory proteins [51]. Except for the host’s control over provisioning precursor molecules (Wilson et al., 2010) [11] and potential regulation of small RNAs [41, 51], no other regulatory machinery has been identified to explain the overall expression of *Buchnera* genes. As the RNA-seq performed in this study was sampled from a wide variety of life stages, the observed variation in BDn gene expression in RWA-SA1 and RWA-SAM could not unequivocally be ascribed to any cue, and hence further study is required. It has been previously reported that high gene %GC content correlated with high expression and low %GC content to low expression [38] for *Buchnera* transcripts. Interestingly, in this study there didn’t appear to be any correlation between %GC content and expression (Supplementary Figure S7). Even if the correlation to *E. coli* protein identity were not to hold true, the expected clustering would have had to at least spread ascendingly linearly from Q2 to Q4. Some genes with very low expression values fell in Q3 (high %GC content, high percentage *E. coli* identity) whilst some genes with very high expression fell in Q2 (low %GC content, low percentage *E. coli* identity). It would thus appear that the expression of *Buchnera* genes is being controlled by a yet unknown mechanism and further study is required before any beneficial data can be gleaned from it.

### Regulation of *Buchnera* transcripts may be due to polyadenylation

Although the detection of *Buchnera* transcripts in aphid RNA-seq experiments is not uncommon [52–54] and has been previously identified in *D. noxia* [33], it is interesting that no further investigations proceeded as to why *Buchnera* transcripts were found to be present after sequencing of a poly-A RNA selected library. The low level of RNA-seq reads that mapped to the BDn reference genome (roughly between 2 and 5%; Additional File 5) was found to be consistent between sequencing libraries constructed through use of total RNA [36] and mRNA capture (this study). It has previously been found that RNA-seq reads obtained from isolated bacteriocytes only contained ~ 33.4% *Buchnera* reads [55] indicating that their total contribution to the mRNA pool of whole body extracted RNA would be minimal. An alternate explanation for the presence of bacterial transcripts in a poly-A selected RNA sequencing library, is the prominent tracts of homopolymeric runs of adenines that the GC poor *Buchnera* genome is well known for [56]. Genic areas containing these tracts may act as anchors for the poly-dT primers during poly-A RNA selection, and thus amplify RNA that do not contain polyadenylated tails. A quick investigation of the number of genic polyA (and inversely also polyT) runs contained in genic areas in BDn revealed that only 65 genes contained runs of 10 bases or more (data not shown), which would thus preclude their presence as possible priming sites. No published data could be found that would indicate any other reason why bacterial transcripts would be present in a poly-dT selected mRNA library.

Polyadenylation in bacteria is not uncommon though as *E. coli* possesses two genes that are involved in polyadenylation, namely poly(A) polymerase (PAP) coded for by the *pcnB* (nicotinate phosphoribosyltransferase) gene and *pnp* (polynucleotide phosphorylase) [57, 58]. Unlike in most eukaryotes, the polyadenylation in *E. coli* is not ubiquitously applied to all mRNA transcripts and the total detectable level of total polyadenylation of *E. coli* has been previously determined to fall well below 10% of all transcripts [59]. *pcnB* is the responsible enzyme for polyadenylating transcripts with long poly(A) tracts where *pnp* only adds short poly(A) tracts. The polyadenylation by *pnp* is roughly responsible for 25% of all detectable polyadenylation in *E. coli*, is highly heteropolymeric (favouring A > G > U > C) and is believed to facilitate RNA turnover and removal of damaged or degraded mRNA [59]. *pcnB* on the other hand is responsible for the bulk of detected polyadenylation in *E. coli* and is believed to facilitate gene regulation [60].

As all *Buchnera* investigated in this study lacks the *pcnB* gene (except for BCc that possess a low %GC copy and BCt that contains two split *pcnB* genes; Additional File 1), the most likely candidate for any polyadenylation in *Buchnera* would be the *pnp* gene (which all *Buchnera* in this study contained). The successful amplification of genic regions in *Buchnera*, from a single stranded cDNA library constructed with only 3’ poly-dT_(18)_ primers, adds a level of experimental evidence for the adenylation of *Buchnera* transcripts. The detected polyadenylation in BDn appears to affect at least the majority, if not all, of the transcripts and its level appears to be close to 100% when taking into account the relative number of BDn transcripts mapped with an mRNA generated sequencing library and total RNA generated sequencing library (Additional File 5). This though will have to be followed up in future studies where focus should be placed on confirming the level of polyadenylation, the polyadenylated tail lengths, and the actual nucleotide composition of the polyadenylated tails.

## Conclusion

A comparative analysis of 10 *Buchnera* from different aphid hosts revealed that there is a remarkable maintenance of overall genomic %GC conservation, albeit their varying genome sizes and gene complements. This is made more noteworthy when considering that the overall nucleotide identity between the 10 *Buchnera* genomes only averages out at ~ 59%. Having compared *Buchnera* PCGs with their *E. coli* homologs, the linear relationship between genic %GC content and homolog identity is striking. Genes that can be readily accepted as central to *Buchnera*’s functioning have maintained relatively high %GC contents whilst those that have deteriorated (in comparison to their homologs) have relatively low %GC contents. It was also evident that *Buchnera* PCGs, from all available annotated genomes on the NCBI, have a set %GC content peaking at ~ 26% that may indicate a threshold for their continued maintenance. Interestingly, the size discrepancy between *Buchnera* genes and their identity to their *E. coli* homologs is quite low, especially when considering the genomic reduction *Buchnera* has undergone. Another first report is the presence of multiple *Buchnera* strains within a single aphid that reproduces through obligate parthenogenesis. As some of the variable SNPs have been maintained between biotypes with documented genealogy, namely RWA-SA1 and RWA-SAM, their maintenance stretches over hundreds of generations. This would indicate that there is likely a fitness advantage to their preservation as their random uptake into developing embryos could ultimately lead to the loss of either strain through genetic drift. The ability to obtain amplified products from a poly-dT_18_ constructed cDNA library, in both PCR and NGS libraries, would indicate that *Buchnera* transcripts are polyadenylated, which may explain the lack of regulatory control regions in the *Buchnera* genome.

## Material and methods

### Assembly of the *Buchnera aphidicola* genome from *Diuraphis noxia*

High molecular weight DNA was extracted from four South African *D. noxia* biotypes (RWA-SA1, RWA-SA5, RWA-SAM and RWA-SAM2) using the Qiagen Gentra Puregene Tissue kit and used for library preparation for three pass CCS sequencing on the PacBio Sequel II system using the HiFi sequencing protocol. Sequences obtained from each sample were then separately assembled through use of the Canu [61] and Hifiasm assemblers [62] making use of the default parameters of each assembler. Through use of quickmerge (with an overlap of 5 000)[63] the Hifiasm assemblies (which were selected as the base assemblies) were merged with the Canu assemblies to produce four separate whole genome assemblies. Contigs representing the uninterrupted genomes of *Buchnera aphidicola* for all four biotypes were then identified through BLASTn comparisons against the genome of *B. aphidicola* from *Acyrthosiphon pisum* (NCBI reference: NC_011833).

Annotation of the assembled *B. aphidicola* genome from *D. noxia* (denoted BDn) was performed separately through the NCBI’s prokaryotic genome annotation pipeline [64]. Areas with high sequence similarity to known genes, but with an interrupted coding frame, were labelled as pseudogenes and their coding domain sequence (CDS) annotation removed, while. tRNAscan-SE 2.0 [65] was utilized to identify tRNAs.

### Comparative analysis of ten *Buchnera aphidicola* genomes

Of the 93 fully sequenced *Buchnera* genomes (from 65 aphid species) available on the NCBI (date accessed 2024/01/12), nine genomes from three aphid subfamilies were selected for comparative analysis (five members from the subfamily Aphidinae, two from the subfamily Lachninae and one from the subfamily Eriosomatinae) with that of *Buchnera* from *Diuraphis noxia (*BDn*)*, and they were *B. aphidicola* genomes from *Acyrthosiphon kondoi* (Bak; Aphidinae), *A. pisum* (BAp; Aphidinae), *Aphis glycines* (Bag; Aphidinae), *Baizongia pistaciae* (BBp; Eriosomatinae), *Cinara cedri* (BCc; Lachninae), *C. tujafilina* (BCt; Lachninae), *Myzus persicae* (BMp; Aphidinae), *Schizaphis graminum* (BSg; Aphidinae) and *Uroleucon ambrosiae* (BUa; Aphidinae) (Table 1). Whole genome alignments were performed with MAFFT v7.308 [66] and phylogenetic analyses were performed with PAUP v4.0 [67] using maximum parsimony. Mauve [68] was used to align syntenic blocks between the 10 *Buchnera* genomes and *E. coli* strain K12 using the “full alignment” option. BRIG v0.95 [69] was used to visualize the genome of BDn in comparison to that of the other *B. aphidicola* genomes. GC curves of the ten *B. aphidicola* genomes was constructed (using the script GC_content.pl) [70], and genes positionally placed in a gene array (Additional File 1) by comparing their relative genic synteny and through similar gene names. The final positions of protein coding genes (PCGs) were refined by manual curation based on the highest percentage protein identity to genes nearby in the array from pairwise alignments with MAFFT v7.308, as well as reciprocal BLASTp matches to their *Escherichia coli* str. K12 homolog. Gene ontology analysis of *Buchnera* genes were performed with OmicsBox v2.0 [71] through BLASTp analysis against the NCBI’s nr database (date accessed: 2023/07). Clustering of shared genes was performed with Cluster 3.0 [72] utilizing Spearman’s Rank correlation and then visualized with Java TreeView 1.0 [73].

### Single aphid DNA extractions, SNP identification, PCR amplification and sequencing of genomic DNA and the *D. noxia* transcriptome

Trimmed Illumina HiSeq reads from *D. noxia* biotypes RWA-SA1 and RWA-SAM [35], and RWA-US2 (GCA_001465515.1; Supplementary Table S1), as well as PacBio HiFi reads from RWA-SA1, RWA-SA5, RWA-SAM and RWA-SAM2 (PRJNA1019137) were individually mapped to the BDn reference genome (NZ_CP013259.1). SNP calling was performed using Geneious 9.1.8 [74], where variants were predicted within the mapped reads excluding the reference (minimum coverage, quality and variant frequency respectively at × 100, Q20 and 20%), and PCR primer design using Primer 3 (Supplementary Table S4) [75].

Genomic DNA was extracted from single apterous *D. noxia* aphids in triplicate (*n* = 3) for several South African biotypes (RWA-SA1, RWA-SA2, RWA-SA3, RWA- RWA-SA4, RWA-SA5 and RWA-SAM) through use of the DNAZol kit (Thermo Scientific) using the manufacturer’s protocol. These were then used as templates in PCRs, along with a pooled DNA extraction of RWA-US2 aphids, to confirm in silico identified SNPs through Sanger sequencing (ABI3730xl) at the Central Analytical Facilities, Stellenbosch University.

Trimmed RNA-seq reads obtained from RWA-SA1 and RWA-SAM (Additional File 5) [37] were mapped to the BDn reference genome (NZ_CP013259.1) through use of HISAT2 [76] making use of the default parameters. The obtained SAM files were converted to BAM files with SAMtools [77] and used to quantify the expression of genes using StringTie [78] using default parameters. The prepDE.py script from StringTie was then used to estimate read counts from the mapping coverage and differential expression (DE) was then calculated through use of edgeR (Additional File 5) [79].

### Supplementary Information

**Additional file 1. **Ordered gene array of the 10 *Buchnera* genomes considered in this study along with measured genomic metrics.

**Additional file 2. **Multiple linear regression modelling of *Buchnera* protein coding genes %GC and length compared to *Escherichia coli*, str K12.

**Additional file 3. **Genes identified as up and down regulated in Chong et al., 2019 were allocated to their respective quadrants (Supplementary Figure S1) for the *Buchnera* genomes considered in the current study.

**Additional file 4. **Polymorphic sites identified in BDn when mapped with the NGS reads obtained from sequencing various *D. noxia* biotypes.

**Additional file 5. **All RNA-seq reads obtained from *Diuraphis noxia* mapped to BDn.

**Additional file 6: Supplementary Table S1.** Results obtained from maping Illumina reads to the reference genome BDn with NGS genomic reads obtained from sequencing *D. noxia* biotypes SA1, SAM and US2. **Supplementary Table S2.** Distance matrix of whole genome alignment of 10 *Buchnera aphidicola* genomes using MAFFT. **Supplementary Table S3.** Genes identified as split from all *Buchnera* considered in this study.** Supplementary Table S4.** PCR primers utilized for confirmatory sequencing of *Buchnera* genotypes and their characteristics. **Supplementary Table S3.** PCR primers utilized for amplification of *Buchnera* transcripts from an oligo dT(20) cDNA library and their characteristics.

**Additional file 7: Supplementary Figure S1. **A Mauve alignment generated from aligning the 10 *Buchnera* *aphidicola* genomes considered in this study along with *E. coli* strain K12.

**Additional file 8: Supplementary Figure S2. **Scatter plot produced by plotting *Buchnera* protein coding gene %GC content over *Buchnera* protein identity towards their *Escherichia coli* protein homologs. 

**Additional file 9: Supplementary Figure S3. **A line graph illustrating the relative percentage of all *Buchnera* genes, from annotated genomes available on the NCBI, plotted over their %GC content. The mean of all the %GC contents was plotted in red.

**Additional file 10: Supplementary Figure S4.** Genes that were predicted to be undergoing positive selection (as obtained from Chong et al., 2019) were plotted over their %GC content and protein identity towards *Escherichia coli*, str. K12.

**Additional file 11: Supplementary Figure S5. **Biological process gene ontology terms of the *Buchnera* genes from *Diuraphis noxia* that contained SNPs to the BDn reference genome.

**Additional file 12: Supplementary Figure S6. **A 2% agarose gel loaded with the PCR amplification products of *Buchnera* transcripts that were amplified from *Diuraphis noxia* cDNA generated with oligo dT(18) primers.

**Additional file 13: Supplementary Figure S7. **The relative expression of protein coding genes of *Buchnera aphidicola* (BDn) plotted over their %GC content and protein identity towards *Escherichia coli*, str. K12. High expression genes logCPM > 12; Mid expression genes logCPM < 12 and > 9; Low expression logCPM < 9.

**Additional file 14: Supplementary Figure S8.** A histogram with the number of *Diuraphis noxia* transcripts plotted over their total relative combined expression for *D. noxia* biotypes RWA-SA1 and RWA-SAM.

## Data Availability

The *Buchnera* genomes constructed in this study have been uploaded to the National Center for Biotechnology Information (NCBI) (https://www.ncbi.nlm.nih.gov/) with the accessions CP136766 (BDn of RWA-SA1), CP136765 (BDn of RWA-SA5), CP013259 (BDn of RWA-SAM), CP136764 (BDn of RWA-SAM2). The transcriptome of *D. noxia* biotypes SAM and SA1 are available on the NCBI with GEO accession numbers GSE143502. Data on the plant host response is also available with accession number GSE120267.
