# Supplementary material for: Host-specific co-evolution likely driven by diet in Buchnera aphidicola
Source: BMC Genomics. 2024 Feb 8;25:153. doi: 10.1186/s12864-024-10045-3 (PMC10851558; doi:10.1186/s12864-024-10045-3)
Supplement: Supplementary file 7 — Additional file 7: Supplementary Figure S1. A Mauve alignment generated from aligning the 10 Buchnera aphidicola genomes considered in this study along with E. coli strain K12. [file 12864_2024_10045_MOESM7_ESM.pptx]

## Slide 1
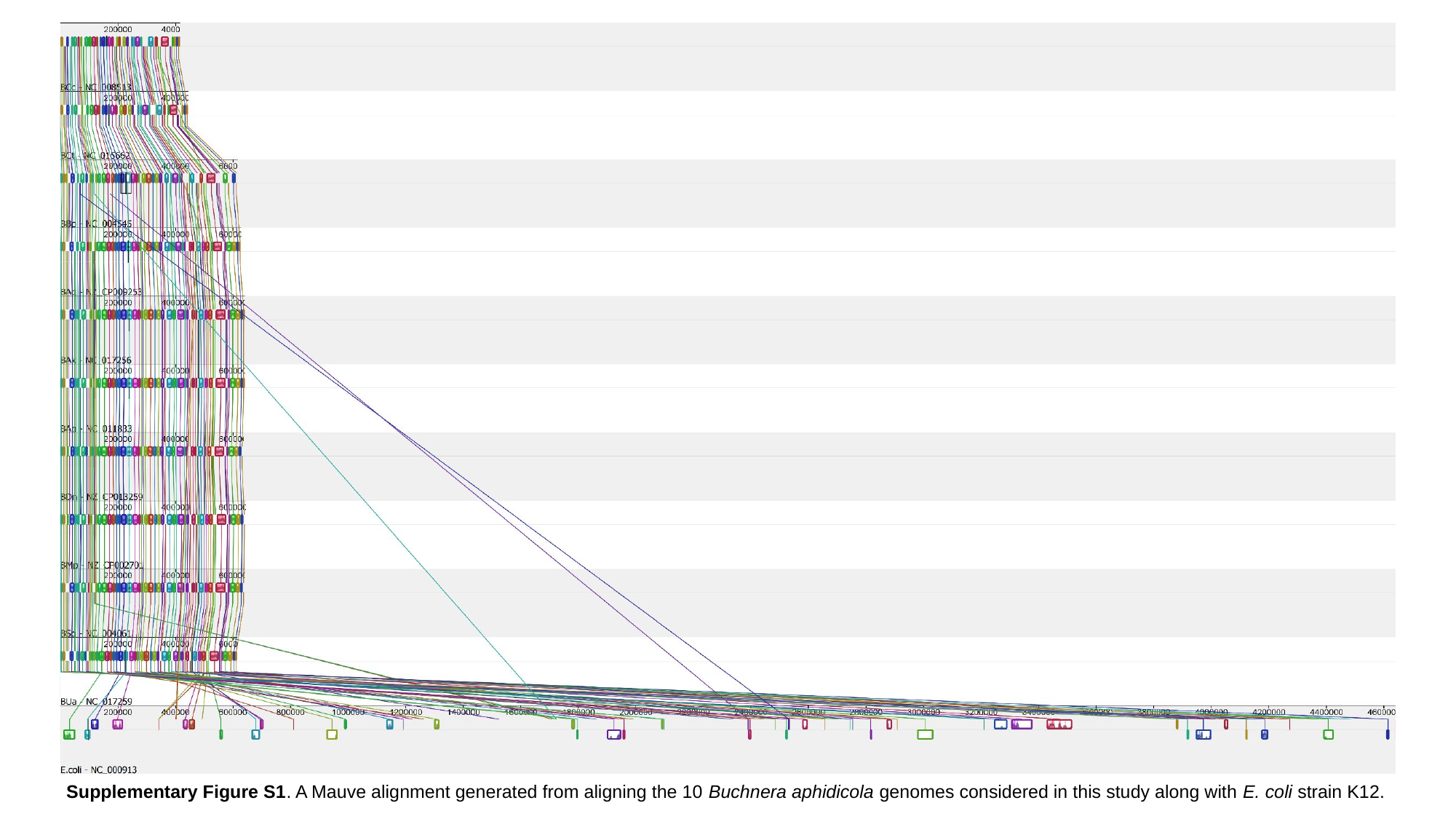

Supplementary Figure S1. A Mauve alignment generated from aligning the 10 Buchnera aphidicola genomes considered in this study along with E. coli strain K12.
