# Supplementary material for: Host-specific co-evolution likely driven by diet in Buchnera aphidicola
Source: BMC Genomics. 2024 Feb 8;25:153. doi: 10.1186/s12864-024-10045-3 (PMC10851558; doi:10.1186/s12864-024-10045-3)
Supplement: Supplementary file 9 — Additional file 9: Supplementary Figure S3. A line graph illustrating the relative percentage of all Buchnera genes, from annotated genomes available on the NCBI, plotted over their %GC content. The mean of all the %GC contents was plotted in red. [file 12864_2024_10045_MOESM9_ESM.pptx]

## Slide 1
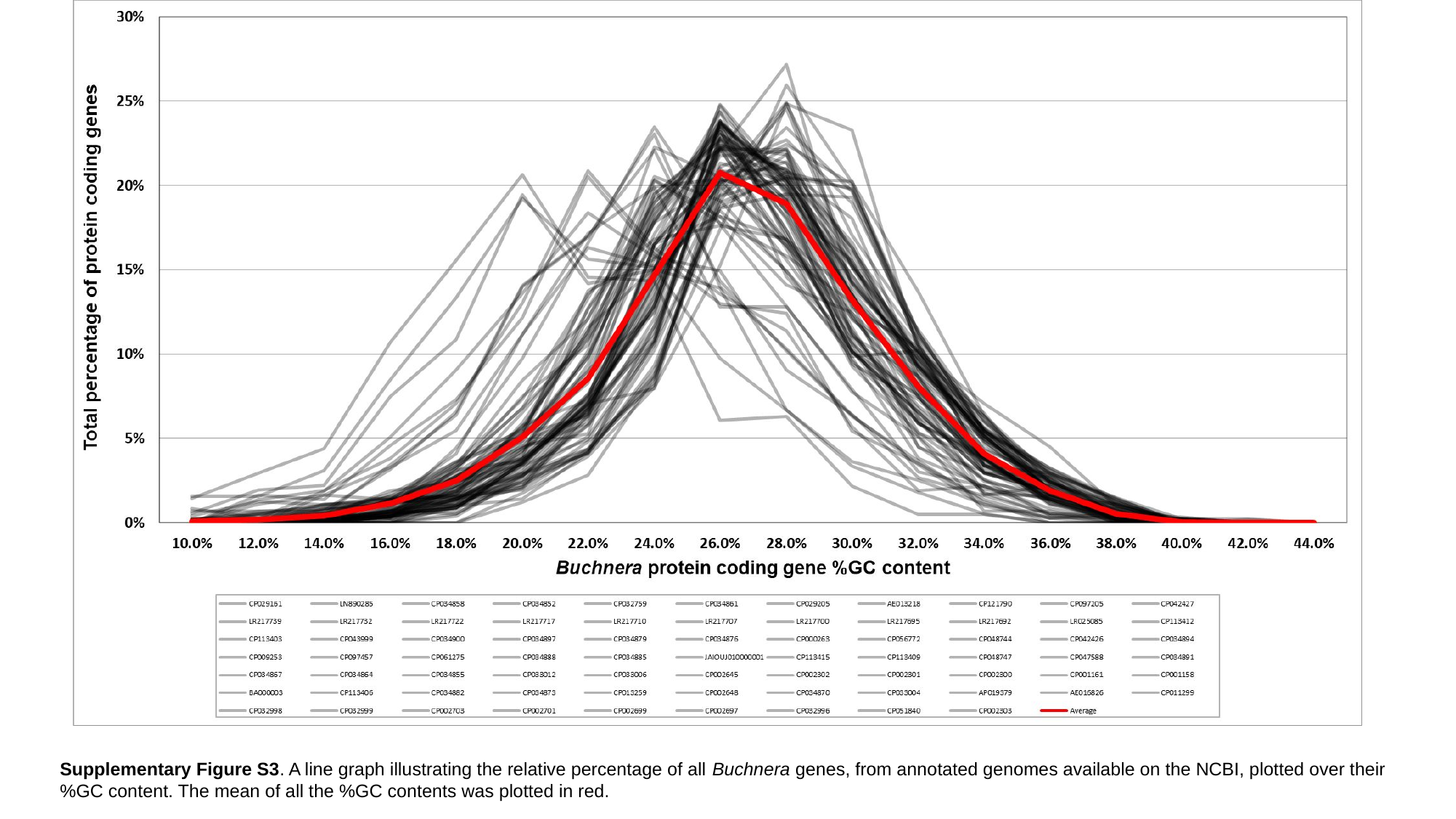

Supplementary Figure S3. A line graph illustrating the relative percentage of all Buchnera genes, from annotated genomes available on the NCBI, plotted over their %GC content. The mean of all the %GC contents was plotted in red.
