# Supplementary material for: Host-specific co-evolution likely driven by diet in Buchnera aphidicola
Source: BMC Genomics. 2024 Feb 8;25:153. doi: 10.1186/s12864-024-10045-3 (PMC10851558; doi:10.1186/s12864-024-10045-3)
Supplement: Supplementary file 10 — Additional file 10: Supplementary Figure S4. Genes that were predicted to be undergoing positive selection (as obtained from Chong et al., 2019) were plotted over their %GC content and protein identity towards Escherichia coli, str. K12. [file 12864_2024_10045_MOESM10_ESM.pptx]

## Slide 1
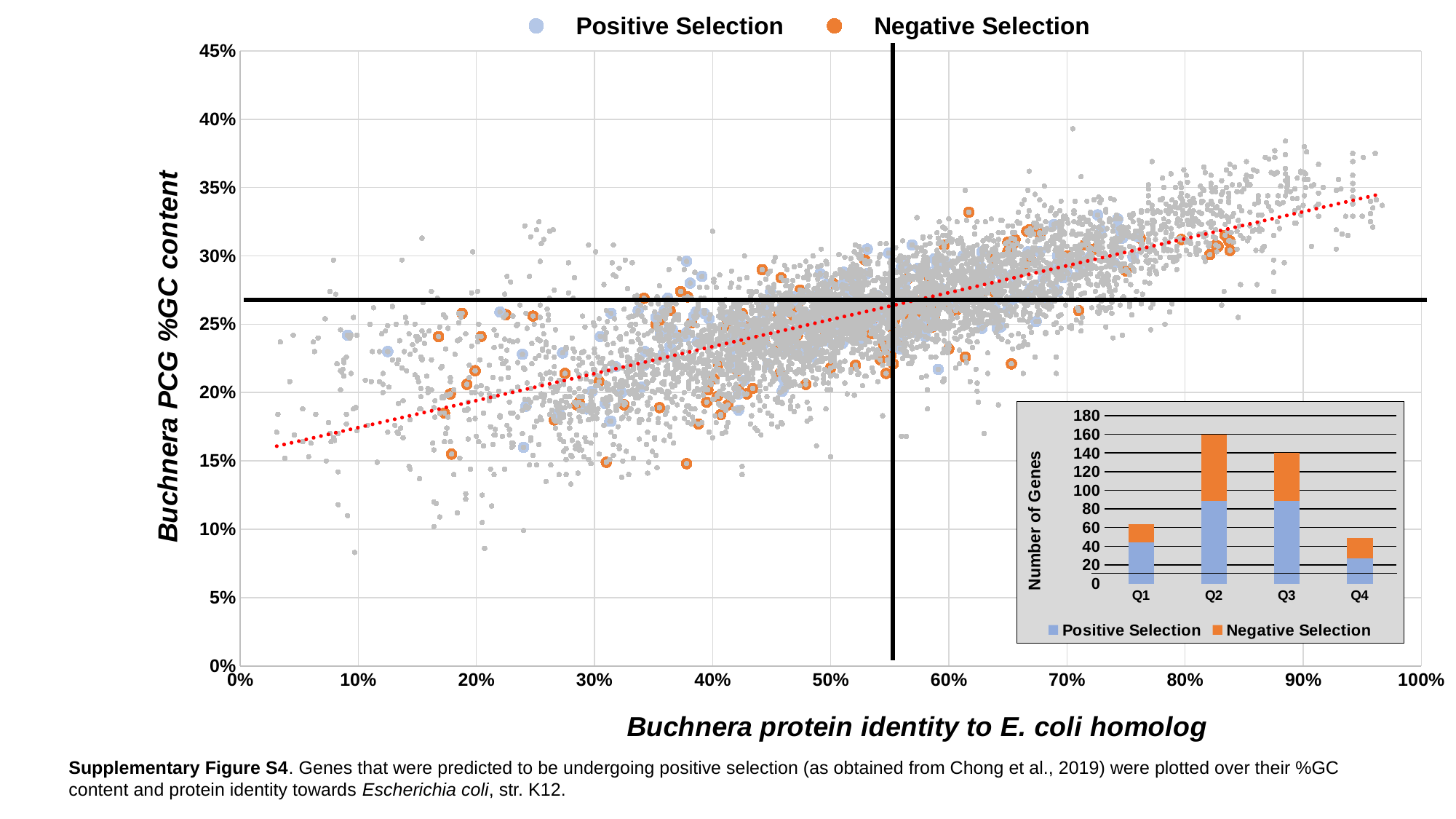

### Chart
| Category | | | |
|---|---|---|---|
### Chart
| Category | Positive Selection | Negative Selection |
|---|---|---|
| Q1 | 44.0 | 20.0 |
| Q2 | 89.0 | 71.0 |
| Q3 | 89.0 | 51.0 |
| Q4 | 27.0 | 22.0 |Supplementary Figure S4. Genes that were predicted to be undergoing positive selection (as obtained from Chong et al., 2019) were plotted over their %GC content and protein identity towards Escherichia coli, str. K12.
