# Supplementary material for: Host-specific co-evolution likely driven by diet in Buchnera aphidicola
Source: BMC Genomics. 2024 Feb 8;25:153. doi: 10.1186/s12864-024-10045-3 (PMC10851558; doi:10.1186/s12864-024-10045-3)
Supplement: Supplementary file 11 — Additional file 11: Supplementary Figure S5. Biological process gene ontology terms of the Buchnera genes from Diuraphis noxia that contained SNPs to the BDn reference genome. [file 12864_2024_10045_MOESM11_ESM.pptx]

## Slide 1
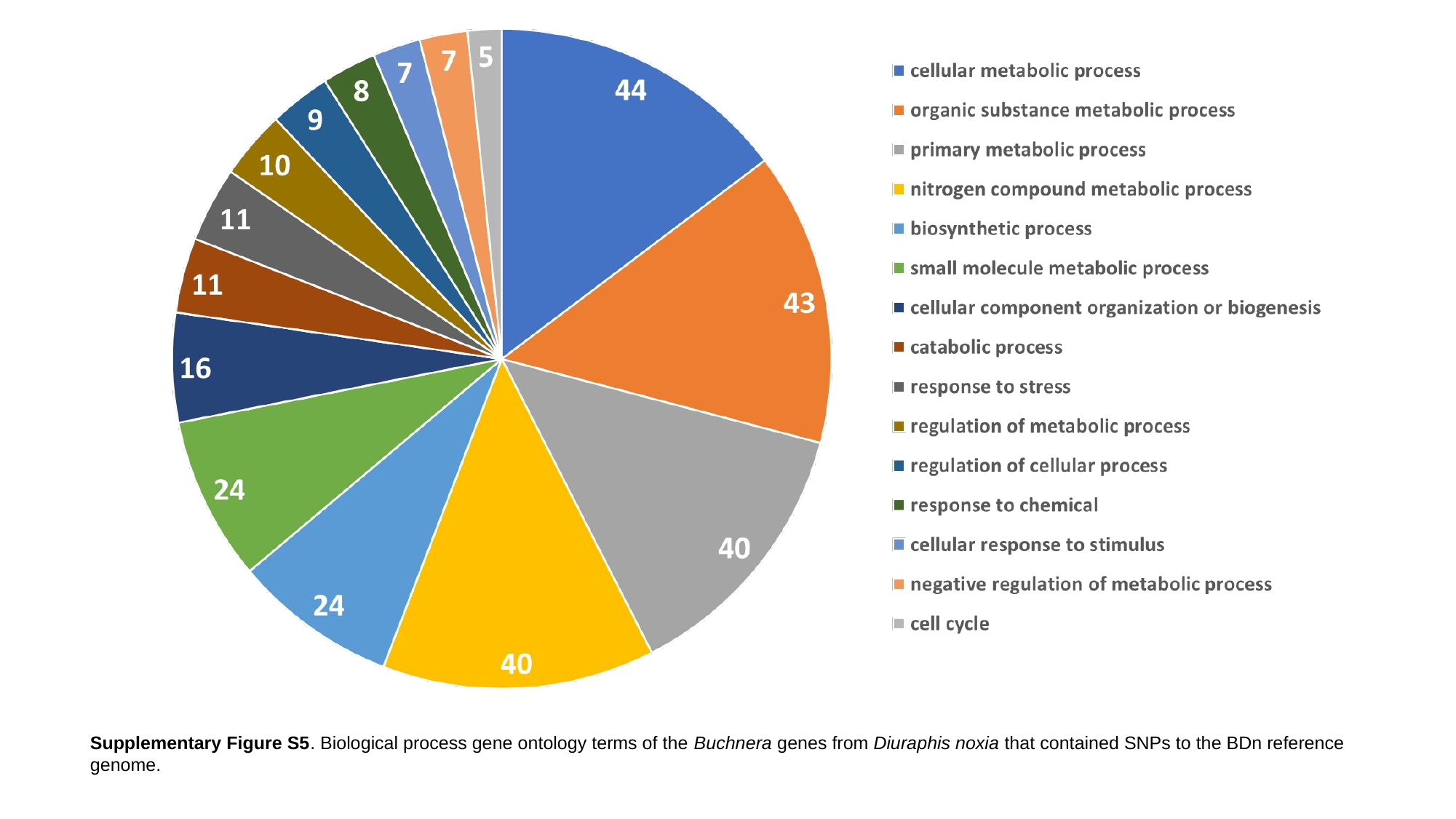

Supplementary Figure S5. Biological process gene ontology terms of the Buchnera genes from Diuraphis noxia that contained SNPs to the BDn reference genome.
