# Supplementary material for: Host-specific co-evolution likely driven by diet in Buchnera aphidicola
Source: BMC Genomics. 2024 Feb 8;25:153. doi: 10.1186/s12864-024-10045-3 (PMC10851558; doi:10.1186/s12864-024-10045-3)
Supplement: Supplementary file 12 — Additional file 12: Supplementary Figure S6. A 2% agarose gel loaded with the PCR amplification products of Buchnera transcripts that were amplified from Diuraphis noxia cDNA generated with oligo dT(18) primers. [file 12864_2024_10045_MOESM12_ESM.pptx]

## Slide 1
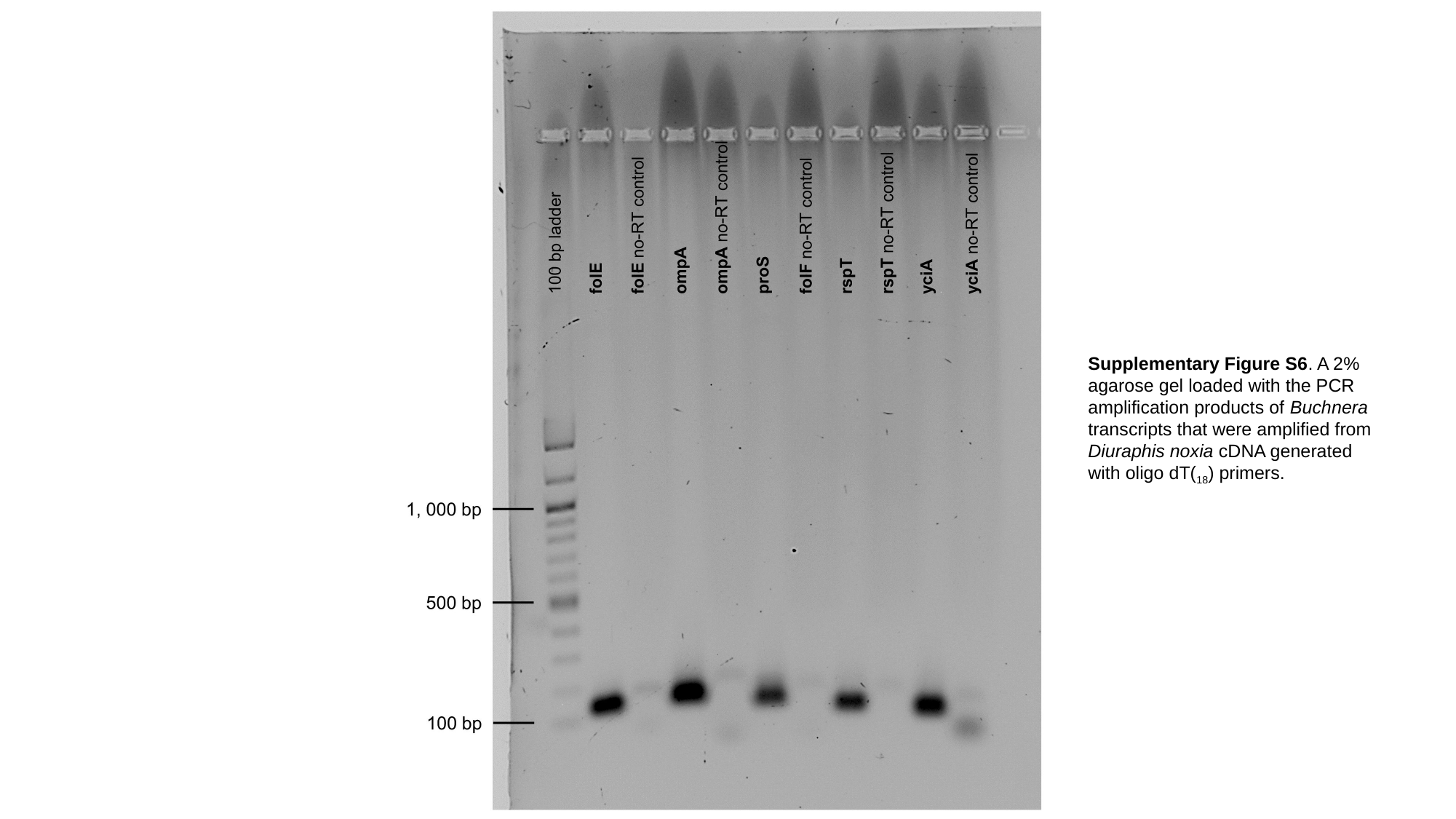

Supplementary Figure S6. A 2% agarose gel loaded with the PCR amplification products of Buchnera transcripts that were amplified from Diuraphis noxia cDNA generated with oligo dT(18) primers.
