# Supplementary material for: Host-specific co-evolution likely driven by diet in Buchnera aphidicola
Source: BMC Genomics. 2024 Feb 8;25:153. doi: 10.1186/s12864-024-10045-3 (PMC10851558; doi:10.1186/s12864-024-10045-3)
Supplement: Supplementary file 13 — Additional file 13: Supplementary Figure S7. The relative expression of protein coding genes of Buchnera aphidicola (BDn) plotted over their %GC content and protein identity towards Escherichia coli, str. K12. High expression genes logCPM > 12; Mid expression genes logCPM < 12 and > 9; Low expression logCPM < 9. [file 12864_2024_10045_MOESM13_ESM.pptx]

## Slide 1
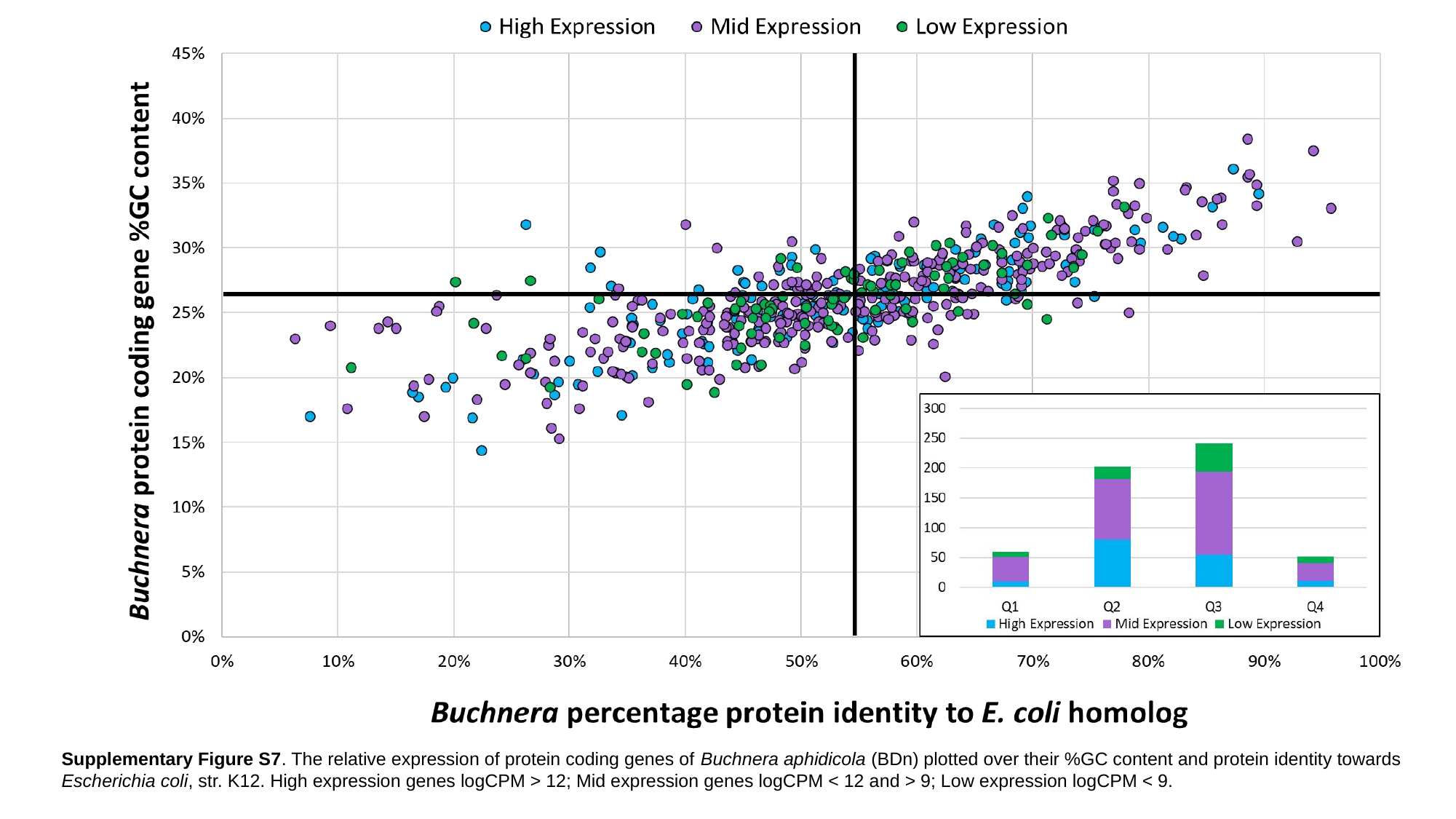

Supplementary Figure S7. The relative expression of protein coding genes of Buchnera aphidicola (BDn) plotted over their %GC content and protein identity towards Escherichia coli, str. K12. High expression genes logCPM > 12; Mid expression genes logCPM < 12 and > 9; Low expression logCPM < 9.
