# Supplementary material for: Host-specific co-evolution likely driven by diet in Buchnera aphidicola
Source: BMC Genomics. 2024 Feb 8;25:153. doi: 10.1186/s12864-024-10045-3 (PMC10851558; doi:10.1186/s12864-024-10045-3)
Supplement: Supplementary file 14 — Additional file 14: Supplementary Figure S8. A histogram with the number of Diuraphis noxia transcripts plotted over their total relative combined expression for D. noxia biotypes RWA-SA1 and RWA-SAM. [file 12864_2024_10045_MOESM14_ESM.pptx]

## Slide 1
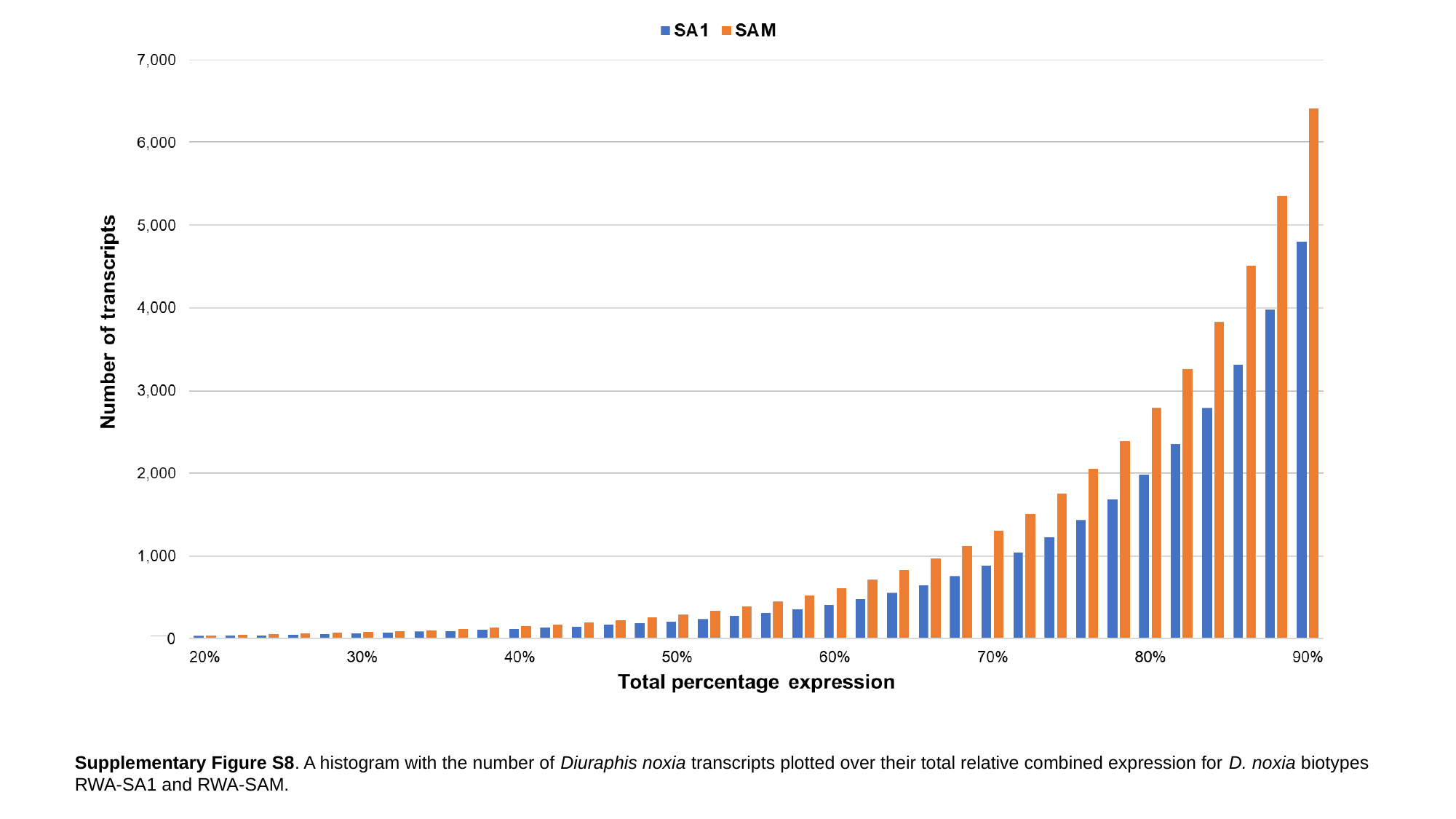

Supplementary Figure S8. A histogram with the number of Diuraphis noxia transcripts plotted over their total relative combined expression for D. noxia biotypes RWA-SA1 and RWA-SAM.
